# A New Cecal Slurry Preparation Protocol with Improved Long-Term Reproducibility for Animal Models of Sepsis

**DOI:** 10.1371/journal.pone.0115705

**Published:** 2014-12-22

**Authors:** Marlene E. Starr, Allison M. Steele, Mizuki Saito, Bill J. Hacker, B. Mark Evers, Hiroshi Saito

**Affiliations:** 1 Aging and Critical Care Research Laboratory, University of Kentucky, Lexington, Kentucky, 40536, United States of America; 2 Department of Surgery, University of Kentucky, Lexington, Kentucky, 40536, United States of America; 3 Department of Physiology, University of Kentucky, Lexington, Kentucky, 40536, United States of America; 4 Markey Cancer Center, University of Kentucky, Lexington, Kentucky, 40536, United States of America; Georgia Regents University, United States of America

## Abstract

Sepsis, a life-threatening systemic inflammatory response syndrome induced by infection, is widely studied using laboratory animal models. While cecal-ligation and puncture (CLP) is considered the gold standard model for sepsis research, it may not be preferable for experiments comparing animals of different size or under different dietary regimens. By comparing cecum size, shape, and cecal content characteristics in mice under different experimental conditions (aging, diabetes, pancreatitis), we show that cecum variability could be problematic for some CLP experiments. The cecal slurry (CS) injection model, in which the cecal contents of a laboratory animal are injected intraperitoneally to other animals, is an alternative method for inducing polymicrobial sepsis; however, the CS must be freshly prepared under conventional protocols, which is a major disadvantage with respect to reproducibility and convenience. The objective of this study was to develop an improved CS preparation protocol that allows for long-term storage of CS with reproducible results. Using our new CS preparation protocol we found that bacterial viability is maintained for at least 6 months when the CS is prepared in 15% glycerol-PBS and stored at -80°C. To test sepsis-inducing efficacy of stored CS stocks, various amounts of CS were injected to young (4–6 months old), middle-aged (12–14 months old), and aged (24–26 months old) male C57BL/6 mice. Dose- and age-dependent mortality was observed with high reproducibility. Circulating bacteria levels strongly correlated with mortality suggesting an infection-mediated death. Further, injection with heat-inactivated CS resulted in acute hypothermia without mortality, indicating that CS-mediated death is not due to endotoxic shock. This new CS preparation protocol results in CS stocks which are durable for freezing preservation without loss of bacterial viability, allowing experiments to be performed more conveniently and with higher reproducibility than before.

## Introduction

Sepsis is a life-threatening clinical condition characterized by a profound systemic inflammatory response to infection. Progression to a more severe condition with shock, multiple organ failure, and death commonly occurs. Sepsis affects more than 700,000 people and claims at least 200,000 lives in the US annually [1]. This disease is particularly serious among the elderly population as both incidence and mortality drastically increase with advanced age [2].

Among various types of animal models for sepsis research, cecal ligation and puncture (CLP) is one of the most frequently used procedures to induce experimental sepsis in laboratory animals such as mice and rats [3], [4], [5]. Under the CLP model, polymicrobial peritonitis is induced in anesthetized animals by surgical ligation of the cecum followed by needle puncture to allow secretion of cecal contents into the abdominal cavity. The CLP model has been preferred by many investigators who study sepsis using laboratory animals because it is a relatively simple surgical procedure and closely mimics the clinical course of intra-abdominal sepsis [3]. The severity of CLP-induced sepsis is highly dependent on the degree of infection which can be influenced by volume, rapidity, and duration of cecal content released into the abdomen, and by the bacterial flora present in the cecum. Therefore, despite its popularity, CLP may not be preferable for certain experiments including those investigating animals with different cecum size, shape, or bacterial flora. This concern may apply for studies that compare severity of sepsis among animals with different body size (ex. neonatal mice or mutant dwarf mice), under different diet regimens (ex. liquid diet, high fat diet, or diet restriction), different gastrointestinal conditions (ex. neonatal or aged animals or animals with gastrointestinal pathology), or increased sensitivity to surgery (ex. aged animals or animals with deficient wound-healing capability).

In cases when CLP is not preferable, intraperitoneal injection of cecal slurry (CS) is an alternative method. In the CS model, contents from the cecum of sacrificed animal(s) are suspended in liquid form and injected into the abdominal cavity of other animals to induce polymicrobial sepsis [6], [7], [8], [9], [10]. The CS model of sepsis has been preferred by a limited number of investigators, particularly those who study sepsis in neonatal mice. However, one prominent disadvantage of the CS model is that the currently accepted protocol for CS preparation with 5% dextrose in water (D5W) does not allow for long-term storage, and thus the CS has to be freshly prepared each time an experiment is performed [11]. This can lead to significant variability from experiment to experiment. In the present study, to circumvent this problem, we developed a new CS preparation procedure that allows for the long-term storage of CS stocks without loss of bacterial viability. In addition, we validated survival rates of mice at different ages using stored CS prepared with this new protocol.

## Materials and Methods

### Animals

Young (4–6 months old), middle-aged (12–14 months) and aged (24–26 months old) male C57BL/6 mice were obtained from the National Institute on Aging. Young (5 months old) mice for diabetes experiments were obtained from The Jackson laboratory. All mice were maintained in pressurized intraventilated (PIV) cages (maximum 5 per cage) in an environment under controlled temperature (21–23°C), humidity (30–70%), and lighting (14 hours light/10 hours dark) with free access to drinking water and chow (Rodent Diet No. 2500, LabDiet, St. Louis, MO). Mice were acclimated for at least 7 days prior to experimentation. All procedures were approved by the Institutional Animal Care and Use Committee at the University of Kentucky and care and handling of animals were performed in accord with the National Institutes of Health guidelines for ethical animal treatment.

### Induction of chronic pancreatitis and diabetes in mice

To induce chronic pancreatitis in mice, recurrent acute pancreatitis was induced in young mice (6-months old) by a procedure modified from our previous protocol for acute pancreatitis [12]. Each mouse received intraperitoneal (i.p.) injection with either physiological saline (control) or caerulein (American Peptide Company, Sunnyvale, CA) at a dose of 50 µg/kg body weight, 6-times hourly, 3 days per week (Monday, Wednesday, and Friday) for 9 weeks. Body weight of each mouse was monitored daily. To induce diabetes, young (5-months old) fasted mice received streptozotocin (Sigma-Aldrich, St. Louis, MO) at a dose of 45 mg/kg body weight, i.p. once daily for 5 consecutive days. Control mice received no injection. Development of diabetes was confirmed by monitoring the non-fasted blood glucose level of each mouse by tail vein nick (ACCU-CHEK Nano blood glucose meter, Roche Diagnostics, Indianapolis, IN).

### Cecum macroscopic analysis, weight, and wet/dry ratio

At sacrifice, ceca were dissected from mice using sterile instruments. Each cecum was placed in a plastic container, weighed, and photographed. The contents of each cecum were collected using sterile forceps and spatula, weighed (wet weight) and dried in an oven at 60°C for 48 h (dry weight). A wet/dry ratio was calculated for the contents of each cecum.

### Analysis of aerobic bacteria in the cecal contents

Immediately after collection, cecal contents from young and aged mice were suspended in sterile water at a concentration of 5 mg/mL. The resulting suspension was mixed well and 50 µL was spread onto multiple agar plates containing 3.7% w/v brain-heart infusion broth (211059, Becton, Dickinson and Company, Sparks, MD) and 0.15% w/v agar (214530, Becton, Dickinson and Company) with 8 mg/L aztreonam (Sigma-Aldrich) for Gram-positive bacteria selection [13], 10^5^ units/L penicillin G (Sigma-Aldrich) for Gram-negative selection, or without antibiotics for total aerobic bacteria quantification.

### Cecal slurry (CS) stock preparation

**Figure 1:**
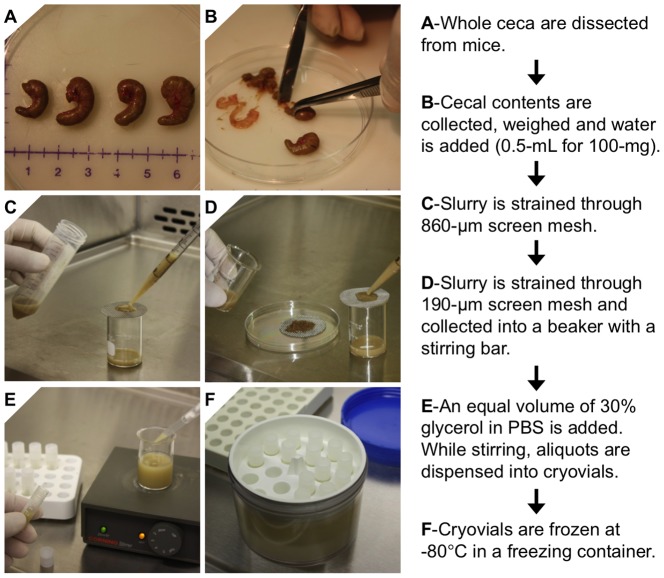
Protocol for preparation of cecal slurry (CS) stock for cryopreservation. Cecal contents were aseptically collected from mice and prepared according to the diagram. An additional filtration step using a 70 µm strainer is recommended after step D.

Four-month-old C57BL/6 mice were sacrificed by cervical dislocation and the whole cecum was dissected from each mouse (Fig. 1A). The entire cecal contents were collected using sterile forceps and spatula (Fig. 1B). The collected cecal contents were combined, weighed, and mixed with sterile water at a ratio of 0.5-ml of water to100-mg of cecal content. This cecal slurry (2xCS) was sequentially filtered through two sterile meshes (first 860-µm and then 190-µm, Bellco Glass, Inc., Vineland, NJ) (Fig. 1 C and D). If visible debris remains, the CS can be further strained through a 70 µm mesh strainer without loss of bacteria. The filtered 2xCS was then mixed with an equal volume of 30% glycerol in phosphate buffered saline (PBS), resulting in a final CS stock solution (1xCS) in 15% glycerol. While continuously stirring on a plate with a magnetic stir bar, 1–2 ml of CS stock was dispensed into cryovials (Fig. 1E). The CS stock aliquots were stored at 4, −20 or −80°C for later comparison. For freezing the samples at −80°C, the CS aliquots were placed in a plastic cryogenic freezing container (Thermo Scientific Nalgene, Waltham, MA) to ensure slow freezing of the bacteria (Fig. 1F).

### Assessment of bacteria viability in CS stocks

Immediately after preparation of CS stock, an aliquot of CS was serially diluted with sterile saline and plated onto multiple agar plates containing 3.7% w/v brain-heart infusion broth and 0.15% w/v agar. CS aliquots stored at various temperatures for different periods of time were also diluted and plated in the same manner. The agar plates were incubated at 37°C in ambient air for 24 hours before colonies were counted to determine colony formation unit (CFU).

### Induction of polymicrobial sepsis using CS

A series of different bacterial doses were administered to freely fed mice to titrate the severity of the model. Mice received various amounts of CS stock by injection into the peritoneal cavity. The frozen CS (kept in 15% glycerol at −80°C) was rapidly thawed by submerging the cryovial(s) in a 37°C water bath, mixed thoroughly, and used immediately for injection with a 21-gauge needle. Rectal body temperature was monitored for each mouse shortly before injection and thereafter at multiple time-points and survival was monitored for at least 10 days. Circulating bacteria were monitored by plating diluted blood taken by tail vessel microsampling from each mouse at 12, 24, and/or 48 h after CS injection.

### Heat-inactivation of CS

To assess the extent to which CS injection induces endotoxemia, heat-inactivated CS was injected into the peritoneal cavity. A CS-containing vial was thawed and subsequently incubated at 72°C for 10 minutes before administration to mice. A portion of the heat-treated CS was diluted and plated onto agar plates to confirm complete bacterial death.

### Statistical analysis

All data are expressed as means and standard deviations. Survival curves were analyzed by Kaplan Meier LogRank test. Other data were analyzed by Student's *t*-test using SigmaPlot Statistical Software version 11.0 (Systat Software, San Jose, CA). A *p* value ≤0.05 was considered statistically significant.

## Results

### Comparison of cecum size, shape, and cecal content consistency in different experimental conditions

**Figure 2:**
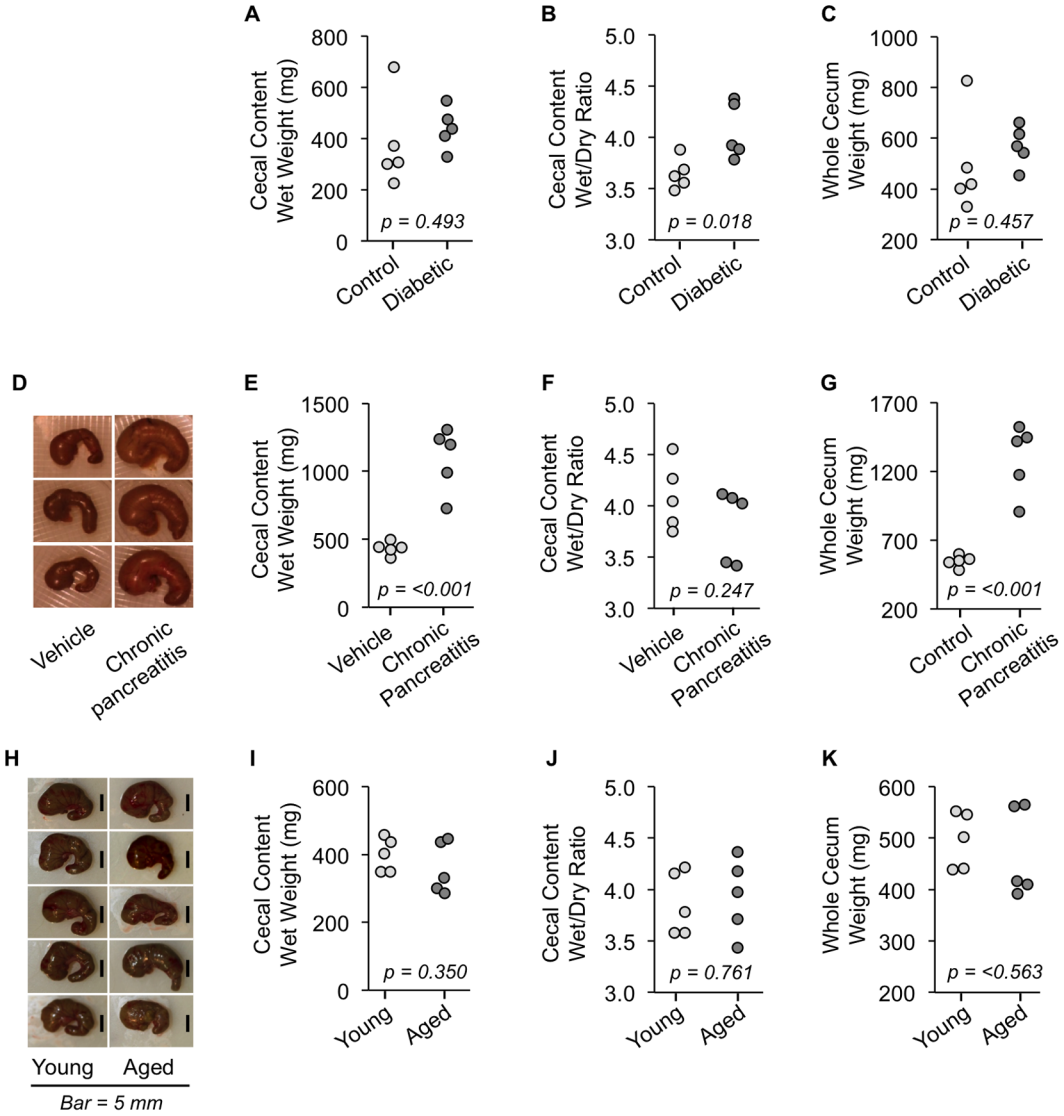
Abnormal cecum characteristics in mice with diabetes, chronic pancreatitis, and aging. Ceca and cecal contents were collected from mice under different experimental conditions. Wet weight of cecal contents (A), wet/dry weight ratio of cecal contents (B), and whole cecum weight (C) from STZ-induced diabetic mice and non-treated control mice (n = 5 each). Macroscopic images (D), wet weight of cecal contents (E), wet/dry weight ratio of cecal contents (F), and whole cecum weight (G) from mice with caerulein-induced chronic pancreatitis and saline-injected control mice (n = 5 each). Macroscopic images (H), wet weight of cecal contents (I), wet/dry weight ratio of cecal contents (J), and whole cecum weight (K) from young and aged mice.

To show that under some experimental conditions the CLP model of sepsis is not ideal, we compared cecum size, shape, and cecal content consistency in various animal models including diabetes, chronic pancreatitis, and aging. Type 1 diabetes was induced by multiple low dose streptozotocin (STZ) injection model (confirmed by hyperglycemia), and ceca were dissected from mice at sacrifice. Macroscopically, the ceca of diabetic and control mice looked similar in size and shape (data not shown). Cecal content wet weight were not different between the two groups (Fig. 2A), but cecal content wet/dry ratio (Fig. 2B) showed a significant difference indicating that fecal matter within the cecum of diabetic mice has a different consistency than normal mice. This could complicate CLP-induced sepsis because the watery cecal content of diabetic mice may leak into the abdomen from the puncture site much more rapidly than that of control mice.

Chronic pancreatitis was induced by repeated bouts of acute pancreatitis using caerulein injection and ceca were dissected from mice at sacrifice. Macroscopically, the ceca of mice with chronic pancreatitis appeared enlarged and fuller than that of control mice (Fig. 2D). Cecal content wet weight was significantly different (p<0.001) between the two groups (Fig. 2E) indicating that mice with chronic pancreatitis have more fecal matter in their cecum. The wet/dry ratio of cecal content between control mice and mice with chronic pancreatitis was not significantly different (Fig. 2F). Whole cecum weight was significantly different (Fig. 2G). These findings indicate that while stool consistency is similar, the enlarged ceca of mice with chronic pancreatitis could be problematic when determining which portion of the cecum to ligate during CLP procedure.

**Figure 3:**
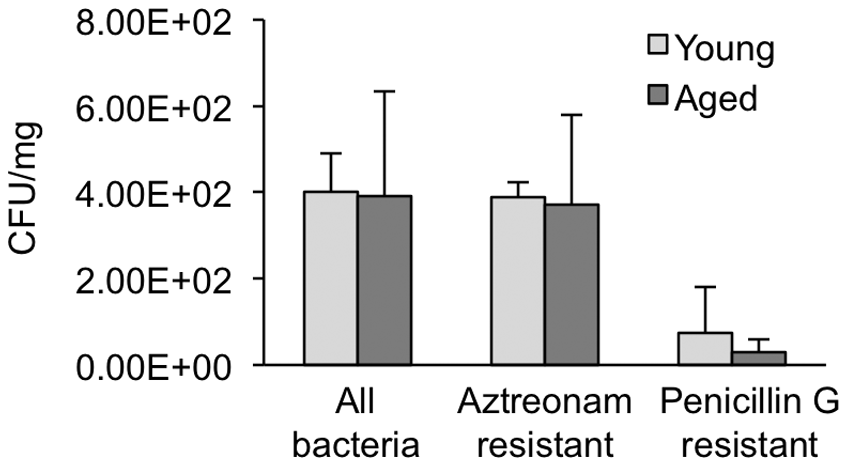
Bacteria quantification of cecal contents from young and aged mice. Cecal contents from young and aged mice were suspended in sterile water and spread onto agar plates without antibiotics, with aztreonam to select for Gram-positive bacteria, or with Penicillin G to select for the majority of Gram-negative bacteria. Data represent the mean ± standard deviation, n = 5 for each group.

We further compared cecum size, shape, cecal content consistency, and bacterial flora in young versus aged mice. The ages of mice used (4–6 months and 24–26 months) are those recommended by aging researchers for gerontological studies because they are representative of the age groups that have clinical presentation of sepsis [2], [14]. Ceca were dissected using sterile instruments from young and aged mice at sacrifice. Macroscopically, the ceca of young mice appear uniform in size and shape, while the ceca size and shape are more variable among aged mice (Fig. 2H). Despite different cecum shape and significantly different body weight in this set of young and aged mice (27.8±2.2 and 36.5±1.7 respectively, p<0.001), cecum wet weight, cecal content wet/dry ratio, and whole cecum weight did not show significant differences between the age groups (Fig. 2 I–K). A small portion (∼100 mg) of the cecal content from each mouse cecum was taken under sterile conditions for aerobic bacterial analysis. Fecal material was dissolved in sterile water at a concentration of 5 mg/mL and spread onto agar plates with penicillin G to select for Gram-negative bacteria, Aztreonam to select for Gram-positive bacteria, or without any antibiotics for total aerobic bacterial quantification. No significant differences were found in aerobic bacteria colony formation for total bacteria, or when selecting for Gram-positive or Gram-negative bacteria (Fig. 3). Collectively, these data indicate that while cecum shape in aged mice are more variable and differ from that of young mice, cecal content in terms of weight and bacterial flora are highly similar.

### Comparison of bacterial viability under different storage conditions

**Figure 4:**
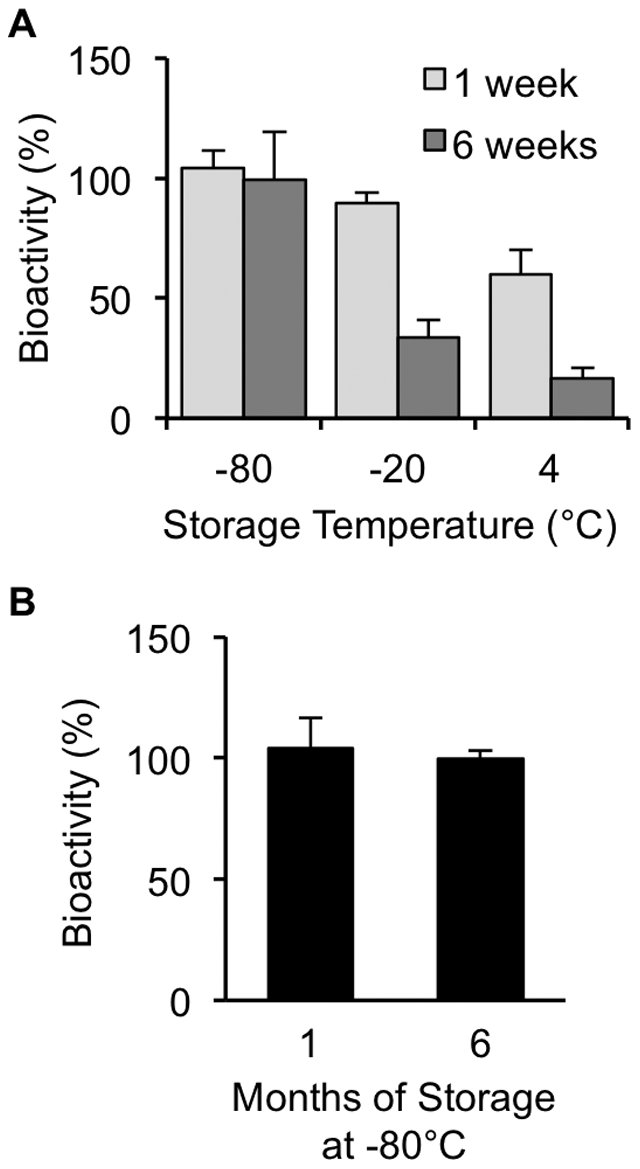
Bacterial viability of cecal slurry (CS) following different storage conditions. Cecal contents from mice were prepared in 15% glycerol and CS samples were stored at −80, −20, and 4°C. Colony formation unit (CFU) was compared one and six weeks later for all storage conditions (A) and up to six months later for samples stored at −80°C (B). Data represent the mean ± standard deviation.

To determine whether bacterial viability could be maintained if stored, CS stocks were prepared in 15% glycerol buffer and stored in aliquots at 4, −20, and −80°C. One aliquot was immediately diluted and spread onto agar plates to determine colony forming ability. One and six weeks after preparation, an aliquot of CS stock from each storage condition was thawed and colony forming ability determined by spreading on agar plates as was done for the freshly prepared solution. Bacterial viability was maintained only in the stocks stored at −80°C (Fig. 4A). Significant loss of viability was observed in the CS stocks at both one and six weeks following storage at −20 and 4°C (Fig. 4A). Similar results were obtained when CS was stored in 5% and 10% glycerol buffer with the colony forming ability maintaining 100% of original capability after cryopreservation at −80°C for 6 weeks (data not shown). Additional CS stocks stored in 15% glycerol/PBS at −80°C were kept for up to six months with bacterial viability being tested periodically. After six months of storage at −80°C, bacterial viability was maintained at 99.5% of the freshly prepared CS solution (Fig. 4B).

### Age-dependent mortality of CS-induced sepsis

**Figure 5:**
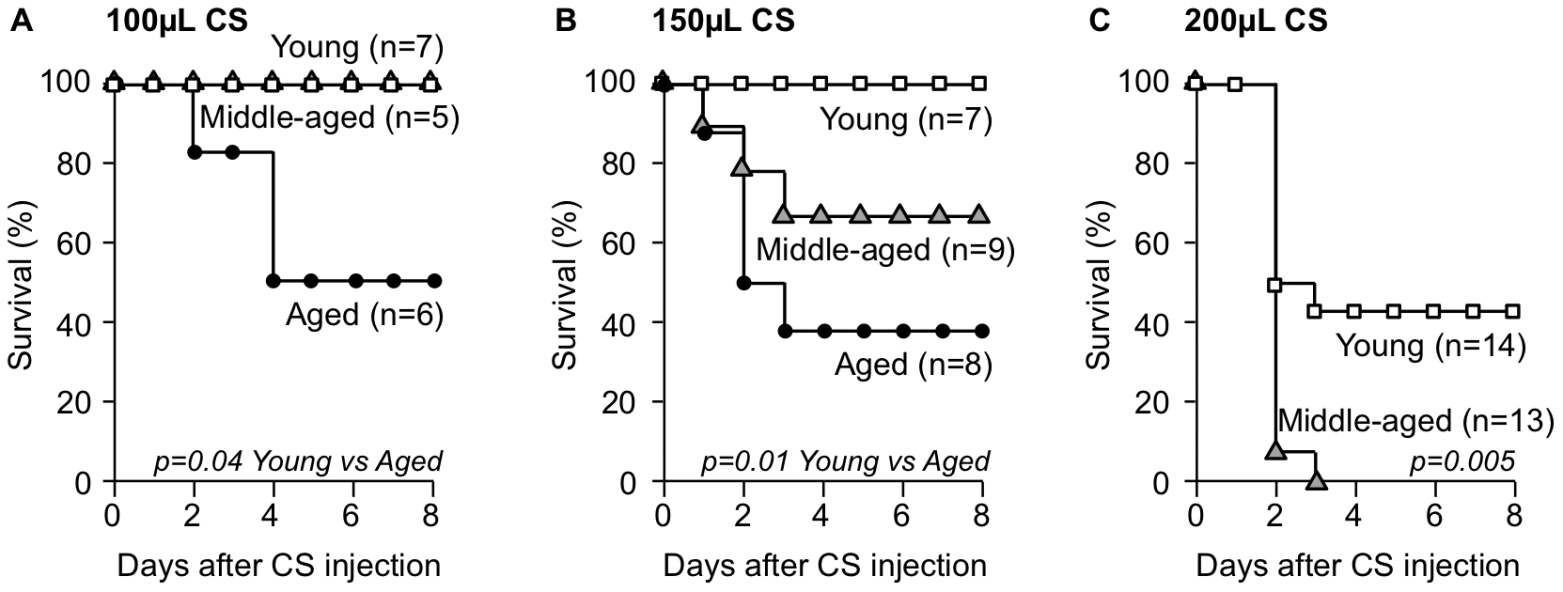
Survival curves of mice after injection with cecal slurry (CS). Young, middle-aged, and aged C57BL/6 mice were injected with either (A) 100 µL, (B) 150 µL, or (C) 200 µL CS and survival monitored for ten days.

To test the efficacy of stored CS stocks for inducing sepsis, three different age groups of mice were intraperitoneally injected with standardized doses of CS and survival monitored for at least 10 days. The CFU of the injected CS was 4.7×10^4^/mL. As shown in Fig. 5A, 100 uL of CS was non-lethal to young and middle-aged mice but resulted in only 50% survival in aged mice. By increasing the dose of CS to 150 uL, survival in aged mice was reduced to 38%, survival in middle-aged mice was reduced to 67%, and young mice maintained 100% survival (Fig. 5B). A dose of 200 uL further reduced survival in aged and middle-aged mice to 0%, while young mice became susceptible with a 43% survival rate (Fig. 5C). Additional doses studied but not shown were 50 uL which resulted in a 63% survival rate in aged mice and 400 uL which killed all young mice.

### Circulating bacteria and mortality

**Figure 6:**
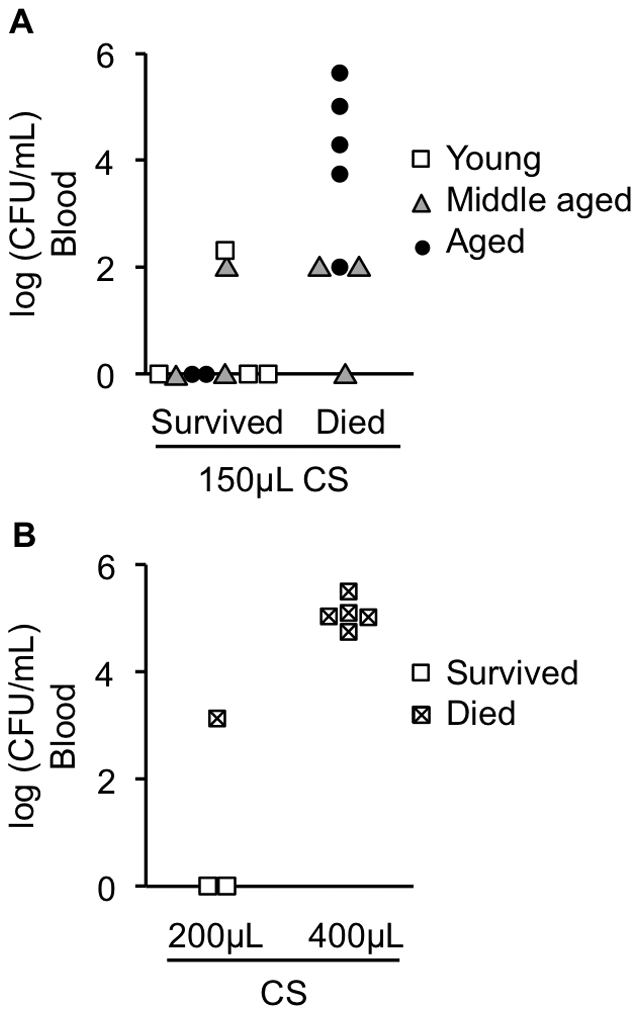
Circulating bacteria amount correlates with mortality. (A) Blood was taken from the tail vein of young, middle-aged, and aged mice 12, 24, or 48 h after injection with 150 µL CS and colony formation unit (CFU) assessed by spreading on agar plates. After fifteen days, data from mice which survived or died were separated into two groups and CFU compared. (B) Blood was taken from the tail vein of young mice 24 h after injection with 200 or 400 µL CS and CFU assessed by spreading on agar plates. After ten days, data from mice which survived or died were separated into two groups and CFU compared. Each symbol represents data from a single animal.

To assess the degree of bacteremia in CS-injected mice, blood was collected from the tail vein of mice and colony formation unit assessed by spreading onto agar plates. Blood was collected at 12, 24, and/or 48 h and the timepoint with the highest CFU was used. After monitoring survival for 10–15 days, blood CFU was compared between survivors and non-survivors. Circulating bacteria CFU correlated with mortality in various ages of mice injected with 200 uL of CS (Fig. 6A) or 400 uL of CS (Fig. 6B). By sampling blood at multiple timepoints, we noticed that the time course of positive bacterial cultures varied from mouse to mouse with some showing positive cultures as early as 3 h and others not until several days after CS injection (data not shown).

### Endotoxemia resulting from bolus CS injection is not responsible for CS-induced death

**Figure 7:**
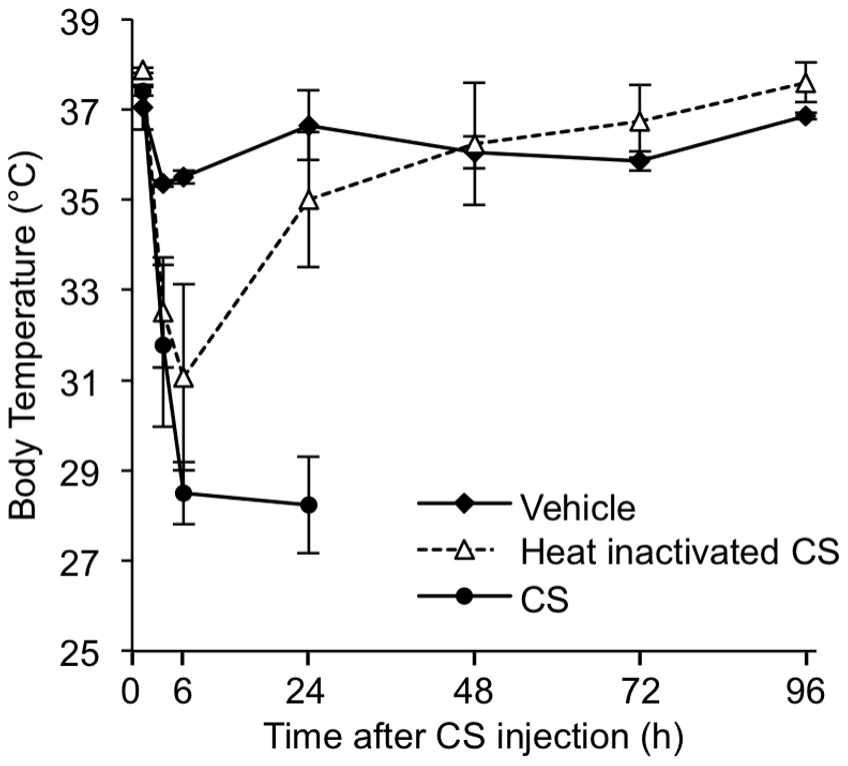
Heat-inactivation of cecal slurry (CS) induces endotoxemia but does not cause mortality after injection to mice. Mice (13-month-old) were injected with 200 µL of untreated CS (in 15% glycerol/PBS), heat-inactivated (72°C, 15 min) CS, or vehicle (15% glycerol) and the body temperatures monitored for several days. CS injected mice died within 48 hours, while no mortality was observed in other groups. Data represent the mean ± standard deviation, n = 3 for each group).

To eliminate the possibility that death due to CS-induced sepsis occurs by endotoxic shock from bolus bacterial injection, an experiment was performed in which CS was heat-inactivated to kill bacteria and then injected to mice (Fig. 7). The same volume of vehicle (15% glycerol) and untreated CS were injected in parallel. Vehicle injection induced a very mild acute drop in body temperature of approximately 1.5°C which returned to baseline within 12 h similar to our previous observation using saline as a vehicle [15]. Both heat-inactivated CS and untreated CS induced profound acute hypothermia with body temperature falling to 32°C within 3 h after injection. Hypothermia was sustained at 32°C in HI-CS injected mice for 24 h at which point the mice began to normalize and all survived, while the untreated CS-injected mice continued to exhibit profound and worsening hypothermia and eventually died. These results suggest that the CS model of sepsis induces non-lethal temporal endotoxemia, but that prolonged illness and death is due to infection.

## Discussion

While performing small animal surgeries or harvesting organs from mice over the last several years [12], [15], [16], [17], [18], [19], we noticed that mice can have highly variable cecum shape and size, and we continuously hypothesized that this variability may affect the result of CLP-induced sepsis. Due to these observations we felt that CLP may not always be an appropriate animal model for inducing experimental sepsis. While others have acknowledged potential problems of the CLP model regarding variability in technique [3], [11], [20], [21], [22], variability in animals used for experiments has been largely ignored. Certainly the benefits of using the CLP model may outweigh the limitations compared to other experimental models of sepsis [3]; however, several characteristics of the animals used in each study design should be carefully considered before assuming that CLP is the ideal model for every experiment. We have shown that the size and shape of the cecum, and/or the nature of cecal contents can be significantly altered by aging or in certain disease conditions (e.g. insufficient digestion in chronic pancreatitis or excessive water consumption in diabetes mellitus). Under these conditions, results from CLP-induced sepsis would be completely misinterpreted, and use of the CS model is a good alternative. For example, CLP performed to compare drug efficacy in mice of the same strain and body size where a subset of the mice receive a drug and another subset do not would be a perfectly appropriate CLP-experiment. While comparing the effects of CLP-sepsis in mice of significantly different body/cecum size (e.g. juvenile vs mature adult, wild-type vs some transgenics), or with significantly different diets or water consumption rates could be problematic due to the nature of the CLP technique.

As aging is one of our research group's primary interests, we took additional effort to characterize the cecum and cecal contents from young versus aged mice. While we did not observe any statistically significant differences in whole cecum weight, or cecal content weight and wet/dry ratio between the ages of mice used in this study (4 month and 24 month), we did notice considerable differences in cecum shape. As can be seen in Figs. 1 and 2, cecum shape can be highly variable, particularly at the distal end (where ligation typically occurs). This variability can alter CLP-sepsis induction since ligation is performed at a designated length, and the shape of the cecum at this location may vary from animal to animal. Our data suggest that if appropriate surgical technique is applied (taking into account variable size and shape of cecum), CLP-induced sepsis is likely in elicit a similar bacterial infection in young and aged mice. Indeed we and others have shown an age-associated increase in mortality after CLP-induced sepsis [15], [23], similar to the current CS study. However, due to sensitivity of aged mice to surgical manipulation [24], CLP may cause other significant responses in addition to the effect of infection. After performing CLP on aged mice, we have also noted multiple times that the large epididymal fat pads of aged mice can adhere to the cecal puncture injury and contain the source of infection which would abrogate CLP-induced sepsis. In our experience this occurred when CLP was performed on older ages of mice (10–26 months of age) with more intra-abdominal adipose tissue, but not in lean young mice (2–4 months of age) with minimal amounts of abdominal fat. This observation is similar to what physicians have observed clinically when the omentum adheres to sites of injury within the abdomen [25]. Though ideal for survival, this occurrence causes a high degree in variability of response depending on whether and when the fat adhered to the cecal puncture in experimental animals.

In addition to other advantages, the use of the CS model is also advantageous in experiments which require large numbers of animals. Experimental sepsis can be easily induced in a large number of animals in a short period of time by CS injection, as opposed to CLP which requires a significant amount of time for anesthesia and surgery on each animal. Likewise, another benefit of the CS model is that surgery is not required, thus potential differences among animal groups in response to surgery or wound healing are circumvented.

Despite some reports indicating the contrary [26], CS is an infectious model. Other groups have supported this notion with evidence of bacterial colonization, abscess formation, systemic inflammation, and spleen alterations characteristic of infection [7]. We further confirmed this using our new CS preparation protocol since analyses of bacteria in the blood in our present study show a strong correlation between circulating bacteria level and mortality. Further, levels of circulating bacteria which exceed the amount of bacteria administered provide evidence of replication. Heat-inactivation of a lethal dose of CS resulted in no mortality suggesting that CS-mediated death results from the infectious component of CS rather than endotoxic shock due to bolus injection. Studies in rats also showed similarity of the CS injection model with human sepsis in that it induces such physiological changes as hypotension, elevated lactate, leucopenia and leukocytosis, and formation of abdominal abscesses [10], [11].

In the preparation of our CS stocks for injection, the cecal contents were suspended in PBS buffer with a final concentration of 15% glycerol before cryopreservation. We used 15% glycerol because it is commonly used for cryopreservation of bacteria [27]. Fecal transplantation studies show 10% glycerol in saline as a suitable suspension buffer for cryopreservation and future administration of fecal matter to patients without loss of efficacy in patient outcome [28]. Recently, we also confirmed that CS stocks can be stored in 5 or 10% glycerol without loss of viability for at least 6 weeks, suggesting that suspending cecal contents in glycerol to a final concentration of at least 5% may also be suitable for CS cryopreservation; however, we have not tested survival curves with these preparations.

A limitation of this study is that we did not fully characterize the bacteria in the CS stocks. We do not know what proportion of aerobic or anaerobic bacteria can survive cryopreservation thus the bacteria remaining in the CS stock after freezing and thawing may not be entirely representative of that derived from freshly injected CS or CLP. However, this does not differ from current CS or CLP studies in which the bacterial flora of mice used is not routinely analyzed and can vary from strain to strain and by vendor [29], [30], [31]. Preparation of CS with our new protocol induces polymicrobial sepsis with organisms endogenous to the host and causes both acute endotoxemia and prolonged bacteremia with the latter being primarily responsible for mortality.

## Conclusions

This new cecal slurry (CS) preparation protocol using glycerol/PBS is durable for freezing preservation at −80°C without loss of bacterial viability, allowing us to perform polymicrobial sepsis experiments more conveniently and with higher reproducibility than conventional methods. Injection of mice with cryopreserved CS can reproducibly induce both mild and severe sepsis with mortality rates dependent on both injection dose and animal age. Further, CS-induced mortality is due to bacterial infection, not acute endotoxic shock since (1) heat-treatment of an otherwise lethal dose of CS did not kill the mice, and (2) there was a strong correlation between mortality and blood bacteria counts.
